# Protective Role of Vitamin D Therapy in Diabetes Mellitus Type II

**DOI:** 10.7759/cureus.17317

**Published:** 2021-08-20

**Authors:** Christine M Zakhary, Hiam Rushdi, Jaafar A Hamdan, Kerolos N Youssef, Aafreen Khan, Mohammed A Abdalla, Safeera Khan

**Affiliations:** 1 Internal Medicine, California Institute of Behavioral Neurosciences & Psychology, Fairfield, USA; 2 Medicine, American University of Antigua, St. John, ATG

**Keywords:** vitamin-d, diabetes type 2, diabetic peripheral neuropathy (dpn), diabetic nephropathy (dn), diabetic retinopathy

## Abstract

Diabetes Mellitus type II (DM II) is a worldwide disease with a rapidly growing parallel prevalence and adversities affecting multi-body systems. Hence, it is imperative to treat DM II effectively, maintaining glucose homeostasis to avoid complications such as diabetic nephropathy, peripheral neuropathy, and retinopathy. Vitamin D, among many benefits, has positive outcomes on hemoglobin A1c (HbA1c) control. It aids in insulin secretion and sensitivity. We systematically screened four databases for relevant information; PubMed, Medline, PMC, and Google scholar. Inclusion and exclusion criteria were applied, and quality appraisal was then done using certain checklist tools: Newcastle-Ottawa tool, AMSTAR (A Measurement Tool to Assess Systematic Reviews) checklist, SANRA (Scale for the Assessment of Narrative Review Articles) checklist, and Cochrane bias assessment. Data were collected from 14 articles, of which eight are systematic reviews and meta-analysis, one is a narrative review, five are randomized controlled trials and three are general information about DM II and Vitamin D. In addition, this article evaluates the clinical significance of Vitamin D administration in DM II from a glucose homeostasis perspective, and complications such as nephropathy, neuropathy, and retinopathy. Vitamin D had a clinical positive impact on glucose level, particularly on hemoglobin A1c (HbA1c) reduction, alleviation of diabetic neuropathy and nephropathy symptoms, and hyperglycemia induced-oxidative stress on the retinal cells.

## Introduction and background

Diabetes Mellitus Type II (DM II) is a complex metabolic disease affecting both developing and developed nations. The incidence of DM II is rapidly increasing worldwide, estimated to reach approximately 592 million in 2035 according to The International Diabetes Federation Prediction [1]. Out of 34 million people among the American population suffering from Diabetes Mellitus, about 90-95% are DM II according to the CDC. DM II is induced via cell receptor response resistance to insulin and pancreatic beta (B) cell dysfunction [2]. Among the complications associated with DM II a common one is peripheral neuropathy. This is a crucial consideration as it is the leading cause of diabetic foot and physical disability due to amputation sequelae, if untreated. Other significant complications to be evaluated are retinopathy and nephropathy, leading to possible blindness and end-stage renal disease, respectively. 

Vitamin D, similar to Vitamin A, E, and K, is a fat-soluble vitamin existing in different forms: Vitamin D3 (cholecalciferol) and Vitamin D2. Vitamin D3 is synthesized in the skin under ultraviolet B (UV-B) radiation, whereas Vitamin D2 is derived from the diet and converted to ergocalciferol via UV-B. In the liver, both cholecalciferol and ergocalciferol are converted to 25-hydroxy vitamin D2 and D3. Then, it is transformed into an active form, 1, 25-dihydroxy vitamin D, in the kidneys via 1-alpha-hydroxylase [3]. Vitamin D is responsible for bone metabolism and also facilitates calcium absorption in the gastrointestinal tract. Among the pathologies Vitamin D is associated with, a crucial disease is DM II [4]. 

Vitamin D regulates insulin secretion by binding to Vitamin D receptors present on pancreatic beta (B) cells [5]. Vitamin D works by increasing insulin sensitivity as it augments the expression of insulin receptors through its binding to the Vitamin D response element present in the human insulin receptor gene promoter [6,7]. It also affects fatty acid metabolism in insulin-responsive tissues through the activation of its transcription factor [8], as well as playing a protective role against cytokine-induced apoptosis [9-11]. Hence, there has been an inverse relationship seen between Vitamin D and complications of diabetes mellitus. For example, studies assessing neuropathy revealed that the presence of Vitamin D receptors in neurons and glial cells plays a role in the formation of neurotrophic factors and neurotransmitter synthetase [12]. Many studies have revealed a strong association between Vitamin D deficiency and neuropathic pain in patients with DM II. In diabetic retinopathy, Vitamin D is considered to be an antioxidant protecting the retina from oxidative stress that induces retinal cell damage. As per studies, diabetic retinopathy is higher among patients with DM II with Vitamin D deficiency than with Vitamin D homeostasis [13]. From a nephrology perspective, Vitamin D improves endothelial damage, which ultimately decreases proteinuria and renal fibrosis.

Our systematic review was done to deduce the role of Vitamin D supplements in glycemic homeostasis and its impact on the DM II population. We will focus on how Vitamin D affects different organs and tissues in patients with DM II. Specifically assessing most common complications associated with DM II such as diabetic peripheral neuropathy, nephropathy, and retinopathy for instance, and see if Vitamin D supplements have a protective role. Additionally, we will discuss the mechanisms by which Vitamin D can play a role in both maintaining glucose levels and protecting role against complications of DM II.

## Review

Methods

We conducted a systematic review following the Preferred Items for Systematic Review and Meta-Analysis guidelines (PRISMA) [14].

Data source and strategy

We searched for articles in PubMed, PMC, MEDLINE, and Google scholar. We applied keywords and medical subject headings (MeSH) terms in combination to identify applicable articles on PubMed.

We used the search strategy DM OR Hyperglycemia OR insulin deficiency OR insulin resistance OR "Diabetes Mellitus/complications"[Majr] OR "Diabetes Mellitus/diet therapy"[Majr] OR "Diabetes Mellitus/drug therapy"[Majr] OR "Diabetes Mellitus/physiology"[Majr] OR "Diabetes Mellitus/physiopathology"[Majr] OR "Diabetes Mellitus/prevention and control"[Majr] OR "Diabetes Mellitus/therapy"[Majr] )AND Vitamin D OR "Cholecalciferol/deficiency"[Majr] OR "Cholecalciferol/physiology"[Majr] OR "Cholecalciferol/therapeutic use"[Majr] OR "Cholecalciferol/therapy"[Majr] ) MeSH strategy to identify the relevant articles.

Study selection and eligibility criteria

Once relevant articles were identified, thorough screening was done by two authors: CZ and HR. Titles and abstracts were screened and then full-text studies were assessed for eligibility. Quality appraisal check was done on relevant full-text articles. Only those articles which satisfied the quality appraisal check of 70% were included. We selected articles published, on human species with type II diabetes mellitus, in English within a time frame of 2016 to 2020. Papers on non-human studies and the ones older than five years were excluded. Secondly, patients with no DM II, gestational diabetes mellitus, or diabetes mellitus type I were excluded. 

Inclusion Criteria

Diabetes Mellitus II in the human population, published in English within 2016 to 2020 with no other comorbidities except complications of DM II. In addition, studies with intervention used in the form of Vitamin D supplements were included.

Exclusion Criteria

Studies not included were: those including/discussing diabetes mellitus I, gestational diabetes mellitus, non-diabetic or pre-diabetic patients, an association of comorbidities outside of DM II, studies in non-English languages, non-human studies, articles older than five years, and studies not using Vitamin D as intervention.

Risk Bias Assessment

The following tools assessed the quality of included studies

· Cochrane Bias assessment for Randomized controlled trails

· Newcastle-Ottawa tool for observational studies 

· AMSTAR (A Measurement Tool to Assess Systematic Reviews) checklist for systematic review articles

· SANRA (Scale for the Assessment of Narrative Review Articles) checklist for traditional review articles

Results

Search Outcome

**Figure 1:**
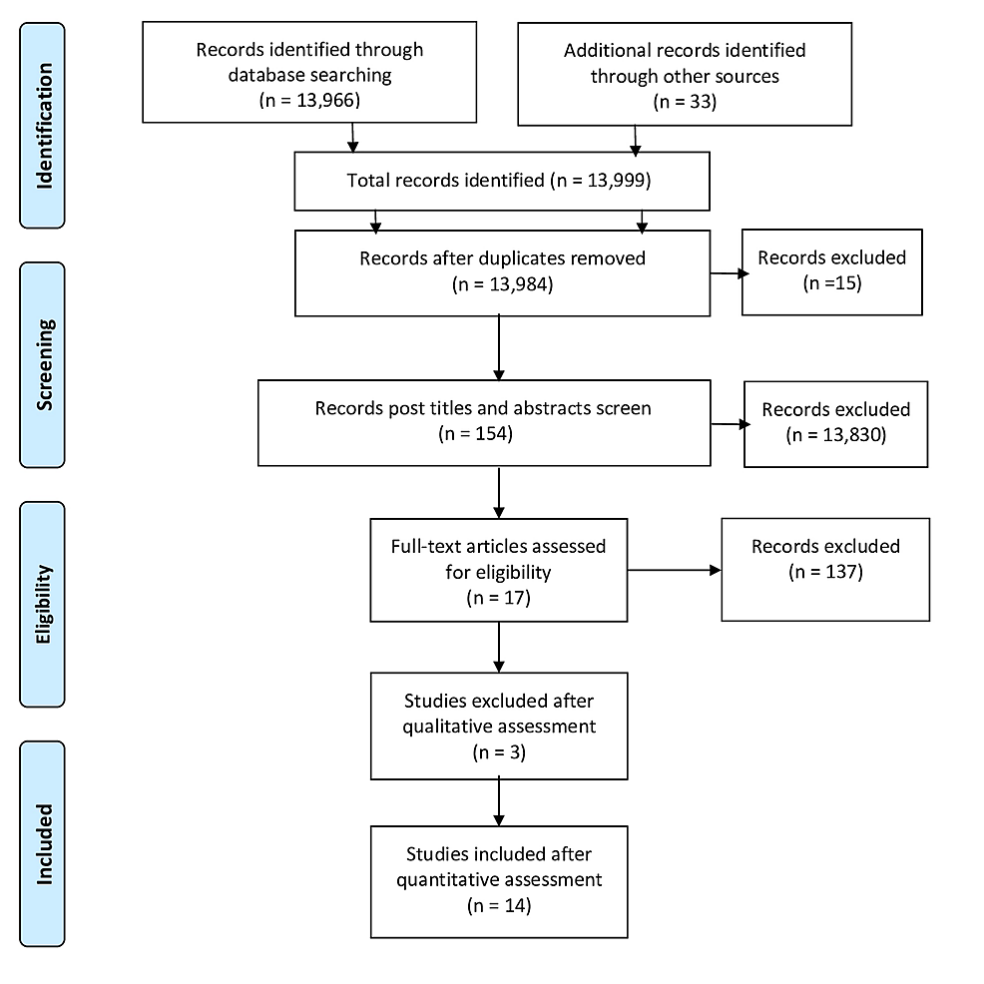
PRISMA flow diagram showing the filtration process for the searched articles PRISMA - Preferred Reporting Items for Systematic Reviews and Meta-Analyses

A total number of 13,999 papers we identified through databases search using MeSH and keywords. Duplicates were removed and a yield of 13,984 papers was obtained. Titles and abstracts were then used as a screening method to rule in/out relevant articles. In addition, eligibility criteria (inclusion and exclusion) were implemented, which provided 17 articles. Post quality appraisal check, the total number of articles used was 14. The 14 articles were: eight systematic review articles, one narrative review, and five randomised controlled trial (RCT) articles. These articles included the effects of Vitamin D therapy on glucose levels, neurons, renal, and retina of type II diabetic patients. In Figure 1, the PRISMA flow diagram displays the article filtering process. 

The first systematic review and meta-analysis collected data from 19 RCTs of 747 patients with short-term Vitamin D supplements. The second meta-analysis collected data from 24 RCTs noting the effect of Vitamin D on HbA1c. Eighteen RCTs assessed fasting blood glucose (FBG); the effect was noticed after subgrouping another RCT of randomly collected patients divided into two groups, one group taking metformin only, the other group metformin with Vitamin D. The result denoted that Vitamin D plays a role in decreasing HbA1c. In the case of neuropathy, we reviewed an RCT of 67 patients with two groups, one treated with 5000 IU and the other 40,000 IU Vitamin D; follow-up was done after 24 weeks, which resulted in high dose Vitamin D supplementation being a factor in improving neuropathy. Regarding nephropathy, reviewing RCT of 1,312 participants, Vitamin D and omega-3 had no significant effect on glomerular filtration rate (GFR). In another RCT, adding Vitamin D to Angiotensin-converting enzyme inhibitors (ACIs) or Angiotensin receptor blockers (ARBs) decreases urinary albuminuria. In an RCT of 85 patients assigned to two groups, the one administered with Vitamin D supplements for six months resulted in a reduction in urinary albumin and an increase in estimated glomerular filtration rate (eGFR), unlike the placebo group. In this systematic review, we focused on the way Vitamin D acts on different organs and its protective role in DM II.

Discussion

Effect of Vitamin D on Glycemic Control

Diabetes Mellitus II is a chronic metabolic disease caused by pancreatic B cell dysfunction or insulin resistance at the receptor level, or both. These conditions are well-thought-out due to chronic inflammation attributable to a high level of inflammatory markers and acute phase proteins [15]. These mediators with adipocytokines produced from inflammation of adipose tissue cause insulin resistance and B cell dysfunction [16, 17]. Vitamin D has anti-inflammatory action, inhibiting cytokine production, which ultimately plays a role in suppressing the state of chronic low-grade inflammation that is present in DM II [18]. Vitamin D enhances the action of insulin through the expression of the human insulin receptor gene that is responsible for providing instructions for the formation of a protein called insulin receptor. Insulin receptor protein is present in the outer membrane of many types of cells and it attaches to the circulating insulin in the blood [7]. This also has a role in enhancing the transcription of the human insulin gene, producing insulin hormone [8]. It also affects fatty acid oxidation through increasing peroxisome proliferator-activated receptor delta (*PPAR-δ*) gene expression [8]. 

Insulin secretion is mediated by several complex processes, the calcium influx and outflux are two among them. Moreover, Vitamin D is the main calcium regulator, which indirectly regulates insulin secretion. Vitamin D receptors are present on pancreatic B cells, enhancing Vitamin D action in response to glucose level to induce insulin secretion [5]. The presence of the 1-alpha hydroxylase enzyme in pancreatic B cells emphasizes its capacity to activate Vitamin D [5]. Calcitriol, the active form of Vitamin D, accelerates the conversion of pro-insulin into insulin. The presence of Vitamin D receptors in skeletal muscle tissue is supposed to play a role in glucose hemostasis in skeletal muscles. Hemoglobin A1c (HbA1c) is defined as non-enzymatic glycosylation of hemoglobin due to hyperglycemia. It is considered the most important test to assess glycemic control over a period not impacted via short-term diabetic agents. A post-meta-analysis review of 19 randomized clinical trials (RCT) assessed 747 diabetic patients with Vitamin D therapy which was compared to 627 placebo patients [19]. It was concluded that short-term Vitamin D supplementation could prevent an increase in HbA1c [19]. 

In another RCT, comprising 140 patients that were divided into two groups, comparing DM II patients taking oral Vitamin D with metformin versus patients taking only metformin was studied; HbA1c results displayed a reduction in the group taking Vitamin D post three months follow-up period [20]. Upon reviewing the meta-analysis, we found that Vitamin D supplement decreases insulin resistance. Similarly, there was an improvement in insulin release [19]. L-type calcium channels present in islets B cells responsible for insulin release are activated by Vitamin D [21]. Fatty acid metabolism is regulated by the peroxisome proliferator-activated receptor delta (*PPAR-δ*) gene, which has a certain role in insulin resistance; hence therapy with Vitamin D decreases insulin resistance [7]. Insulin resistance can be both diagnosed by the level of glucose and insulin secretion in the body. In another meta-analysis of 20 trials of oral Vitamin D supplement, 13 portrayed a reduction in fasting blood glucose; no change was observed except in subgroups like the Middle Eastern population showing a marked reduction. Fifteen trials for HbA1c level showed no significance except in certain obese and a certain cultural background subgroups; seven trials for insulin resistance showed a significant reduction in HbA1c [22].

Vitamin D supplement effect differs among people of different ethnicities. Middle eastern subjects showed best outcomes than Asians due to Vitamin D binding protein polymorphism [22]. People of minimal sun exposure due to wearing certain clothing types are most susceptible to getting Vitamin D deficiency, to whom when given Vitamin D supplementation tend to present with a more significant response to treatment. However, the obstacle is not only the absence of Vitamin D, but it also depends on the antidiabetic agent and diet control.

Effect of Vitamin D on Diabetic Peripheral Neuropathy

Diabetic peripheral neuropathy, a complication of diabetes, is a common nerve disorder that negatively influences the patients' quality of life. It ranges from painless neuropathy to severe pain. It is the leading cause of diabetic foot which can result in limb amputations. Many observational studies revealed an inverse relationship between Vitamin D levels and diabetic peripheral neuropathy, and also found Vitamin D levels to be lower in those with painful diabetic peripheral neuropathy when compared to those without pain [23]. A high dose of intramuscular Vitamin D alleviates the pain, as shown in clinical trials that considered Vitamin D as a potent analgesic [24]. In addition, oral supplementation improves nerve function in addition to its effect in improving wound healing in diabetic foot and ulcers [25]. Vitamin D has a neuroprotective effect as it regulates the neurotrophin level (proteins that induce the survival, development, and function of neurons) and neuronal calcium homeostasis [12]. 

An RCT study of 67 patients of DM II with neuropathy and administration of Vitamin D 40,000 IU weekly revealed improvement of symptoms and decrease in interleukin-6 (IL-6) and elevation of interleukin-10 (IL-10) [26]. Another study revealed that Vitamin D administration results in the reduction of pain caused due to the decrease or even withdrawal of semi-synthetic opioids. An increase of 1 ng/ml of 25-hydroxy vitamin D [25(OH)-D] is associated with increased nerve conduction frequency by 2.2% and 3.4%, respectively [27]. Another study demonstrated that the manifestation of diabetic peripheral neuropathy was more severe when serum 25(OH)-D level is less than 16 ng/ml. An increase in HbA1c is associated with higher tumor necrosis factor-alpha (TNF-alpha) and IL-6, and lower IL-10 [28]. Additionally, Vitamin D is noted to decrease both TNF-alpha and IL-6 and increases IL-10 through immune cells [28]. A significant effect was noticed in patients who were taking high doses of Vitamin D [28]. 

Effect of Vitamin D on Diabetic Nephropathy

The prevalence of chronic kidney disease (CKD), defined as a persistent decrease in GFR or increase in urinary albumin excretion by more than 25%, is increasing in parallel with the duration of diabetes despite the use of therapeutic agents [29,30]. In a meta-analysis, short-term supplements of the active form of Vitamin D3 resulted in a significant reduction in urinary albumin excretion in comparison to placebo; however, long-term treatment has no effect on GFR [31]. In high doses, Vitamin D is suspected of having a reno-protective effect. In an RCT study by Agarwal et al., involving 1, 25 di-hydroxyergocalciferol analogue, it was found that there was a 50% reduction in urinary albumin excretion in CKD stage III and IV with secondary hyperparathyroidism (not undergoing renal replacement therapy) in comparison to 25% in the placebo group; however, this trial included CKD patients with not only diabetic nephropathy but also from other etiologies [32]. In a randomized cross-sectional trial, the use of sufficient Vitamin D3 for 12 weeks in patients with diabetic nephropathy and hyperparathyroidism showed a significant reduction in urinary albumin [33]. Another RCT study showed similar results but was independent of the presence of hyperparathyroidism, and most of the participating patients having GFR above 30 ml/min were not Vitamin D deficient [34]. A study by Li et al. revealed that the active form of Vitamin D has an inhibitory effect on the renin-angiotensin system as it suppresses renin release, and therapy with Vitamin D could help in preventing hypertension [35].

Effect of Vitamin D on Diabetic Retinopathy

The retina is exposed to oxidative stress due to its high content of polyunsaturated fatty acids and high uptake of oxygen and glucose oxidation which ultimately is the main cause of diabetic retinopathy [36]. Oxidative stress is defined as a state of imbalance between antioxidants in the body and free radicals, leading to tissue damage. Hyperglycemia causes oxidative stress in both direct and indirect approaches. The direct effect is induced through auto-oxidation during glucose metabolism [37]. Whereas the indirect effect is through advanced glycation end-products (AGEs) [37]. AGEs target retinal blood vessels, causing stiffness and decreasing elasticity. AGEs also activate nitric oxide (NO) synthase and through diacylglycerol trigger Protein Kinase C (PKC) and nicotinamide adenine dinucleotide phosphate (NADPH) oxidase, generating reactive oxygen species (ROS) [38]. Reduced concentration of low molecular weight antioxidants like glutathione and inactivation of antioxidant enzymes like catalase and superoxide dismutase (SOD) play a major role in diabetic retinopathy as there is no way to overcome oxidative stress caused by hyperglycemia [39].

Vitamin D can enhance the expression of antioxidant enzymes and other enzymes involved in ROS detoxification, such as SOD, glutathione peroxidase (GPx), and glutathione reductase (GR) [40]. This promotes the production of both reduced glutathione and GR [40]. It also up-regulates superoxide dismutase 2 (SOD-2), which is a major mechanism by which Vitamin D protects against oxidative stress [40]. It is worth mentioning that, it was noticed in DM II studies that there is an inverse relationship between Vitamin D level and activity of GPx and GR, and a positive relationship with SOD activity when compared to control [40]. An increase in Vitamin D level enhances the level of glutathione, hence the antioxidant pathway, acting as an immunomodulator through preventing translocation of nuclear factor-kappa B that inhibits inflammatory reaction and apoptosis [41]. Vitamin D and vitamin D receptors (VDR) control the respiratory activity of mitochondria and preserve their integrity to protect against oxidative stress. Hence, Vitamin D supplementation may prevent the progression of diabetic retinopathy, but it is still under evaluation. However, in general, Vitamin D has a positive effect on blood glucose levels and on the most common complications of DM II as portrayed in Table 1.

**Table 1:** Summary of the effect of Vitamin D supplement on various tissues/organs in Diabetes Mellitus II patients VDR - Vitamin D receptor; HbA1c - Hemoglobin A1c

Organs/Cellular Processes/ Tissues	Effect of Vitamin D
Pancreas	Increases insulin secretion through its activation in pancreatic B cells, enhances the transformation of pro-insulin into insulin.
Fatty acids	It enhances fatty acid oxidation, decreasing insulin resistance.
Skeletal Muscles	Through the expression of VDR in skeletal muscles, it is supposed to have a role in glucose hemostasis.
Kidney	Decreases urinary albuminuria.
Nervous system	Improves nerve conduction. Potent analgesic effect.
Skin	Improves wound healing through improving skin microcirculation.
Hemoglobin A1c (HbA1c)	Decreases HbA1c.
Retina	Protective against oxidative stress.

Limitations of study 

Collecting data from English published sources limits the inclusion of possible additional data on the use of Vitamin D. This might cause exclusion of certain data relevant to Vitamin D use in DM II patients published in different languages. Hypervitaminosis D limits the use of Vitamin D in DM II patients with normal Vitamin D levels. Another major limitation of this study is the inclusion of data published between 2016 and 2020 resulting in the exclusion of important, if any, studies and data published before 2016 and after 2020. Moreover, this study focused primarily on DM II patients and excluded patients with diabetes mellitus I, gestational diabetes, and pre-diabetes which could have possibly been used to assess the benefits of using Vitamin D therapy. 

## Conclusions

In this systematic review, the role of Vitamin D supplements and their effect on glucose control and most common complications was evaluated in diabetes mellitus II patients. It was concluded that Vitamin D acts in numerous ways on different systems, and its receptors present in many organs. Upon reviewing several studies, the role of Vitamin D was found to be beneficial in DM II for controlling blood glucose level and hemoglobin A1c level. It increases both insulin sensitivity and insulin secretion. Vitamin D suppresses the inflammatory state in DM II, which in diabetic peripheral neuropathy is crucial. Also, Vitamin D improves nerve conduction and hastens wound healing in cases of diabetic foot. Moreover, it acts as a potent analgesic. In case of diabetic nephropathy, it is very important to maintain its control as it can lead to end-stage renal disease. It was noted that in post-Vitamin D administration urinary albumin excretion decreased. The retina is vulnerable to elevated blood sugar as it causes the release of reactive oxygen species, leading to retinal tissue damage. Vitamin D is a potent antioxidant, stimulating antioxidant enzyme to prevent damage caused by such oxidative stress. Diet control and antidiabetic agents are the cornerstones in controlling blood glucose which can slow any disease-associated complication. A future investigation of this systematic review would possibly investigate Vitamin D administration in having positive preventative outcomes on DM II-related complications. Moreover, it is worth examining if Vitamin D should be given to only Vitamin D deficient patients or all diabetic patients.
